# Health and Social Care Reform in Scotland – What Next?

**DOI:** 10.5334/ijic.5633

**Published:** 2021-10-29

**Authors:** Anne Hendry, Maimie Thompson, Peter Knight, Eleanor McCallum, Alison Taylor, Helen Rainey, Andrew Strong

**Affiliations:** 1 University of the West of Scotland, GB; 2 University of Stirling, GB; 3 Public Health Scotland, GB; 4 Health and Social Care Scotland, GB; 5 Scottish Government, GB; 6 The Health and Care Alliance Scotland, GB

**Keywords:** integrated care, policy, transformation, evaluation, reform

## Abstract

**Introduction:**

This paper analyses the important enablers, barriers and impacts of country-wide implementation of integrated health and social care in Scotland. It offers insights for other systems seeking to advance similar policy and practice.

**Description:**

Landmark legislation was based on a shared vision and narrative about improving outcomes for people and communities. Implementation has involved coordination of multiple policies and interventions for different life stages, care groups, care settings and local context within a dynamic and complex system.

**Discussion:**

Relational and citizen led approaches are critical for success, but it takes time to build trusting relationships, influence organisational and professional cultures and cede power. Assessing national impacts is challenging and progress at a national level can seem slower than local experience suggests, due in part to the relative immaturity of national datasets for community interventions. Five years on there are many examples of innovation and positive outcomes despite increasing demographic, workforce, and financial challenges. However, inequalities continue to increase.

**Conclusion:**

Realising the true value from integration will require a stronger focus on place-based prevention and early intervention to achieve a fairer Scotland where everybody thrives. Solidarity, equity, and human rights must guide the next phase of Scotland’s story.

## Introduction

Integrated care is advocated as an effective way to improve outcomes for people with chronic disease or complex needs [1,2]. However, much of the evidence is based on small scale studies or in specific care groups [3,4,5,6,7] and evidence of country wide large scale system change is lacking [8,9,10]. Recently, several regions have applied realist evaluation [11] and designed mixed methods studies to understand what works in scaling up integrated care [12,13,14,15,16].

This article reflects on progress of large scale, system wide reform of health and social care in Scotland since 2015. It does not purport to be an independent academic evaluation. Rather, it presents the experience of expert stakeholders in developing and implementing national policy for integrated health and social care and a critique of the related enablers, barriers, and impacts. The content draws on a documentary review and analysis of published reports conducted in 2019 by the International Foundation for Integrated Care’s hub in Scotland (IFIC Scotland) for the European Union funded Scirocco Exchange project (*https://www.sciroccoexchange.com/*). Synthesis was undertaken by experts with different perspectives drawn from IFIC Scotland’s multi-sector Reference Network *https://integratedcarefoundation.org/ific-scotland-3*. Thus, it blends both the reference and reflective quality paradigms of integrated care research [17].

Discussion of enablers and barriers is framed around the nine conceptual pillars proposed by the International Foundation for Integrated Care [18]. These pillars map to the building blocks for integrated care developed by the European Commission [19] and operate across the macro, meso and micro levels of integrated care [20]. The impacts of system change are based on publicly available national and local data that relate to the Quadruple Aim of improved population health, enhanced quality of care, better care and provider experience, and more cost –effective care [21].

### Country Context

As a devolved nation within the United Kingdom (UK), the Scottish Parliament has legislative powers across a wide range of policy areas including health and social care [22]. Policy for the National Health Service (NHS) in Scotland is the responsibility of the Scottish Government and funding is resourced through a block grant from the UK Treasury. Fourteen territorial NHS Boards are responsible for planning and providing healthcare for their local population, accountable to Scottish Ministers, the Scottish Parliament and ultimately the electorate. The Convention of Scottish Local Authorities (COSLA) provides political leadership on behalf of Scotland’s 32 local councils who directly provide or commission a wide range of services including social work and social care. Around 80–85% of local authority funding comes from the Scottish Government in the form of a block grant.

The Scottish population is projected to grow from approximately 5.4 million in 2014 to 5.7 million by 2039 [23]. By then, one in four people (25%) are expected to be over 65 years compared with 14% in 1983 [24]. The prevalence of chronic disease in Scotland increases with age, from 25% of adults aged 16–24 to 77% of those aged 75 and over [25]. Around one in five people (20%) in Scotland have multimorbidity and its prevalence is 38% higher in the most deprived decile compared to the least deprived areas [26]. The onset of multimorbidity occurs 10–15 years earlier in deprived areas compared with more affluent areas, particularly multimorbidity that includes a mental health disorder [27].

#### Description of the Policy Reform

Integrating health and social care has long been a policy ambition in Scotland [22,28]. Faced with demographic, workforce, and financial challenges, in 2011 the Christie Commission [29] proposed four priorities for reform of public services:

- A shift towards prevention and early intervention to prevent negative outcomes.
- Greater integration of public services at a local level bringing public, third and independent sector partners together with communities to deliver outcomes that matter to people.
- Greater investment in the people who deliver services through developing the workforce and effective public sector leadership.
- Improved performance through greater transparency, innovation and use of digital technology.

### Laying the Foundations

The Reshaping Care for Older People (RCOP) programme [30], an early policy response to Christie, brokered collaboration between health, social care, housing, third sector and independent providers. Between 2011 and 2015, around 1% of the annual healthcare and social care budget for older people was ring-fenced as a Change Fund to be used for local transformation. The programme helped to generate support for services to be jointly planned, financed, and delivered across the whole system [31]. Highland, in the north of Scotland, was the first area to press for radical reform and a partnership agreement between Highland Council and National Health Service Highland was established in 2012 under existing legislation [32]. Known as the Lead Agency Model, NHS Highland took responsibility for planning, resourcing, and delivering adult health and social care services. In a reciprocal arrangement, the council became the lead for children’s services.

### Nine Pillars to Enable Implementation

#### Shared values and vision

Faced with the scale of the required reforms, by 2011 there was cross party agreement that ambitious new legislation, strategies, and policies would be required [22]. Widespread engagement, both national and local, fostered strong support for a shared vision and values for integration: better health and well-being outcomes through care and support at home and closer to home designed around what matters to people and communities [22,32,33,34]. Macro level engagement with senior leaders from healthcare, local government, housing sector, voluntary organisations, and independent care providers was followed by dialogue with local health and social care organisations, professional bodies, care regulators and trade union representatives. A series of ‘town hall’ conversations across the country heard the voices of local citizens, family carers and the workforce. The extensive dialogue built a movement for change with strong and enduring support for the ‘why’ of integration. However, the details of the ‘what’ and the ‘how’ were less clear.

#### System wide governance and leadership

New legislation to integrate health and social care was introduced through the Public Bodies (Joint Working) (Scotland) Act 2014 [35]. This required shadow arrangements to be in place by April 2015 and governance to fully integrate services by April 2016 through one of two models: Lead Agency (as described for Highland) or a Body Corporate Model. In the Lead Agency model, the lead agency becomes the “Integration Authority” for specific services and the accountability rests with the relevant Chief Executives and Finance Directors. With the exception of Highland [32], all others areas chose a Body Corporate Model where the NHS Board and corresponding local authority (or local authorities where the NHS Board interfaces with more than one local authority) delegate responsibility for planning and resourcing services to new Integration Authorities (IA) [22,28,35]. Thirty IAs were established as distinct legal entities, each operating under the direction of their Integration Joint Board (IJB) comprising non-executive members of the local NHS Board, elected members from the local authority and clinical, Third sector and community representatives. Two new senior posts of Chief Officer and Chief Finance Officer for each IA provide a single point of management for integrated services and related budget. The absence of an overly prescriptive approach was generally perceived as a virtue, allowing a degree of emergence to agree on the scope and details of the local organisational model.

The Chief Officers have two sets of leadership accountabilities: (i) to the IJB for strategic leadership, and (ii) to the NHS Board and local authority for operational leadership [22,28,35]. Chief Officers and IJB Chairs are responsible for building the effective relationships, collaboration, trust, and openness to challenge that are key requirements for successful leadership and management of integrated care [36,37,38,39]. Effective system leadership for large scale change must be distributed, operating at all levels, and involve people who both use and provide care and support [40,41]. At a macro level, the Ministerial Strategic Group [42] which predated the legislation continues to provide valuable high level national strategic direction and leadership for integration. At the meso level, Health and Social Care Scotland (*https://hscscotland.scot*) was established by Chief Officers as a national learning network to build a social movement for change and to share good practice. However, significant turnover in Chief Officers, IJB Chairs and in senior NHS, local government, and civil servants since 2016 has challenged relational continuity [43,44].

At the point of care delivery, effective integrated care is heavily influenced by culture, trust and relationships between professionals from different teams, care setting and sectors [45,46]. A series of coaching and collaborative action learning programmes have attempted to address these human factors [47]. A new Masters programme ‘Leading People-centred integrated care’ was introduced by the University of the West of Scotland in 2018. Both of these initiatives target mid-career community health and care professionals and partners from third sector and strive to build leadership capability and enable succession planning. However, to date there has been limited engagement of professionals from acute hospitals in the national and local integrated care development programmes. This raises concerns about the need to further strengthen relationships between community and acute services.

#### Aligned payment systems

Recognition of the benefit of understanding the relationships between costs, activity and variation for different population groups to inform joint strategic planning and commissioning led to the development of an Integrated Resource Framework [48]. More than £9.4 billion in health and social care resources are now directed by IAs with approximately 70% of this funding delegated by the NHS and 30% by Local Authorities [49]. However, in 2019, most IAs recorded deficits or requested additional funding from their National Health Service Board, local authority or the Scottish Government [50].

Provision of social care is acknowledged as an important contributor to current overall health and social care cost pressures. Free personal social care for people aged over 65 years, first introduced in 2002 [51] was extended in 2019 to adults with degenerative neurological conditions. For both care groups, domestic services, day care or the accommodation element of care home costs may be chargeable. While Adult Social Care was the focus of a rapid, wide ranging independent review launched in 2020, the report of the review recommends some changes in the membership of IAs and in processes for commissioning services and care [52].

#### People as partners in care

A strong focus on what matters to people and communities has been central to policy on integration in Scotland [22,31,34,35]. Efforts to embed personal outcomes in practice have largely focused on re-orienting conversations at the point of care to achieve outcomes identified through shared decision making [53,54,55]. New integrated Health and Social Care Standards seek to improve personalisation and outcomes across all health and care providers [56]. Empowerment, co-production and personalisation are further supported by legislation that places a statutory duty to offer choice in how people are assessed and receive their social care or support, [57] and by a human rights-based Carers Charter and legislation [58]. IAs are tasked with involving the public, people who use services, politicians, and professionals in local service redesign. There are examples of good practice in engagement and co-design but also concerns over examples of tokenistic consultation and limited public involvement [32,44,59,60].

#### Resilient communities and new alliances

Scottish policy has a strong commitment to outcomes and asset-based approaches [61,62]. Health and Social Care Scotland’s Statement of intent signalled a commitment to develop new alliances to create more sustainable compassionate and caring communities [63]. The Long Term Conditions Alliance, established in 2009 as a national intermediary for a range of health voluntary organisations, was reformed as the Health and Social Care Alliance Scotland (the ALLIANCE *https://www.alliance-scotland.org.uk/*) to amplify the voice of over 3,000 voluntary sector organisations and associates as partners for integration. Their analysis of progress in fostering new alliances cited continuing organisational, cultural, and financial challenges [64] but also many examples of successful asset-based initiatives [65]. Examples include: Community Links Practitioners [66] who work alongside primary care to support people living in challenging circumstances or facing loneliness, mental health problems, addictions or debt to draw on individual and community assets; strength based collaborative care and support planning [67]; national and local support for self-management [68]; and social prescribing initiatives [69].

#### Workforce capacity and capability

Integration has not changed the regulatory framework or accountabilities for professional practice, but national guidance describes clinical and care governance for integrated working [70]. The National Workforce plan [71] set out commitments to develop the right workforce skills and capacity. Implementation of this plan is largely work in progress, but a critical step was the introduction of a new General Medical Services contract for general practitioners [72], supported by additional investment in primary care of £250 million to 2021/2022 [73]. The new contract introduces new roles in primary care for community mental health professionals, community link workers, pharmacists, and advanced nursing and allied health practitioners to improve access and quality of care for individuals and communities [74]. The independent review of social care is expected to make some recommendations on workforce capacity and capability, the vital role of the third sector, and the ‘wicked’ issue of parity of pay and conditions between social care and healthcare workers [52].

The vital contribution of improvement support for large scale change cannot be overstated. Between 2006 and 2016, improvement support for integrated working was provided by the Joint Improvement Team, a multi-sector improvement partnership overseen by senior representatives of the Scottish Government, NHS Scotland, COSLA, Third Sector, Independent providers and the Housing Sector [31,34]. From 2016, in an attempt to rationalise improvement support for health and social care, the Scottish Government commissioned the established national healthcare scrutiny and improvement organisation, Healthcare Improvement Scotland, to extend their portfolio to integrated health and social care. With hindsight, changing well established implementation support relationships to IAs may have been an additional challenge at a time when relational continuity, trust and organisational memory were critical. Examples of improvement in primary care and community services are reported in several publications [39,44,75] and at *https://hscscotland.scot/resources/*.

#### Digital solutions

Scotland’s Digital Health and Care Strategy [76] promotes technology enabled care solutions such as Home and Mobile Health Monitoring, Near Me Video Enabled consultations, Digital Platforms and Telecare initiatives. A Strategic Portfolio Board provides oversight of investment and support and has engaged advice from global experts, industry and academia [77]. The digital health and care delivery programme helped build readiness for rapid innovation and adoption of digital solutions in response to Covid-19. Notably an improvement approach underpinned the unprecedented scale up of Near Me video enabled consultations [78]. The approach has been well received by patients, carers, family and professionals [79].

#### Population health and local context

The places we live in and the wider determinants of health have a powerful impact on outcomes [80,81]. The Scottish Index of Multiple Deprivation 2020 [82] provides granular data on these determinants for data zones of around 800 people in 6,976 neighbourhoods. The interactive tool can be used by IAs to identify where people experience disadvantage across different aspects of their lives in order to target health and care resources to local areas with greatest need. However, understanding of population health and prioritisation of targeted investment for specific localities remains relatively underdeveloped. Investment in local analytical expertise and population health management data and tools is supporting leads for strategic planning and commissioning to better meet the needs of local populations [83]. Data Sharing Agreements specify who can get access to data, for what purpose, and set out the process for authorisation and any restrictions [84].

#### Transparency of progress, results, and impact

Published reports note positive progress in collaborative working and encouraging evidence of impacts, albeit with significant local variation in the pace and scale of progress [39,42,44,64,65]. Since 2017, national scrutiny bodies have undertaken detailed joint inspections in eight IAs (27%) to review leadership, performance and strategic planning and commissioning processes and outcomes. Scotland’s National Performance Framework [85] describes the outcomes and indicators that track progress in achieving Scotland’s national purpose, values and ambitions. IAs produce annual reports on indicators for nine national health and wellbeing outcomes [86]. These indicators draw on routinely recorded hospital and community data and on regular national surveys of care experience. National health and care data and data linkage systems offer ways to measure the impact of the reform, in particular through analysis of key trends over time. To appreciate these trends in the context of an ageing population, it is possible to consider how trends over the decade might have looked (‘expected’) in the absence of transformational change.

**Figure 1:**
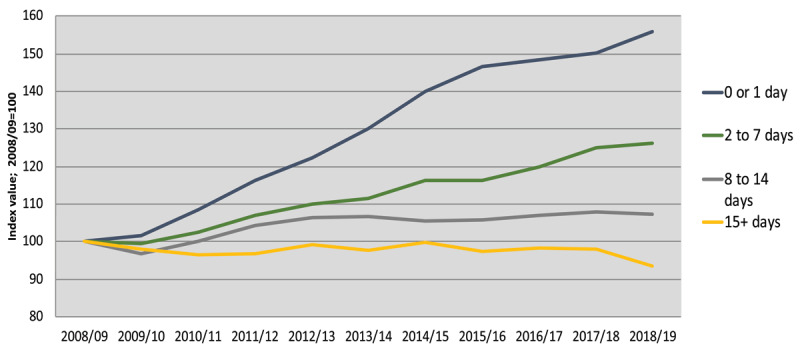
Emergency admissions by average days stay 2008/09 to 2018/19. Source: SMR01 (PHS).

Emergency hospital admissions, a sentinel system level indicator, have risen annually [87]. The trends for people aged 65 and over admitted as an emergency, indexed to 2008/09 (prior to the RCOP programme) and presented by broad length of stay, are shown below (***Figure 1***). The analysis reveals a 56% increase by 2018/19 in the number of older people admitted for urgent assessment who return home within hours or after one overnight stay. The trend for stays of between 8 and 14 days rose by only 7% over the period with virtually no change since 2012/13. The number of older people staying 15 days or more has been largely static since 2008/09, whilst decreasing slightly in 2018/19.

**Figure 2:**
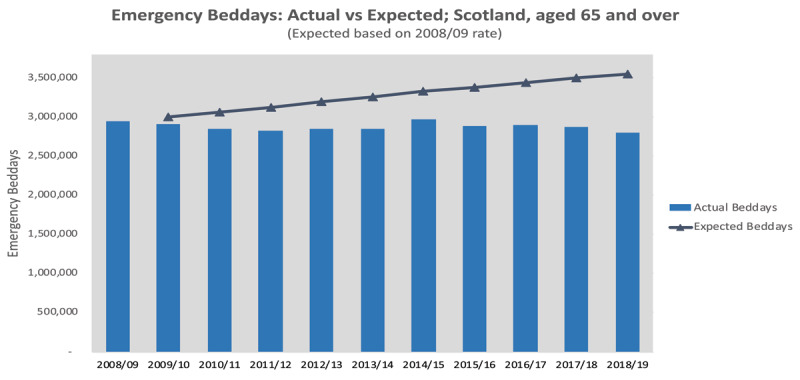
Emergency beddays used by people aged 65+, 2008/09 to 2018/19: actual vs expected. Source: SMR01 (PHS), NRS population estimates.

Further perspective on changes over the decade can be gained by comparing an ‘expected’ trend, adjusted for the changing age profile of the population since 2008/09, alongside actual trends. The first example here shows such a comparison for use of acute hospital beds by older people following emergency admission. The gap between the actual and the expected use of beds (‘emergency beddays’) has increased year by year since 2008/09. The number of emergency beddays used during 2018/19 (2.8 million) is considerably less than ‘expected’ (3.5 million) based on projection of the 2008/09 rate (***Figure 2***).

**Figure 3:**
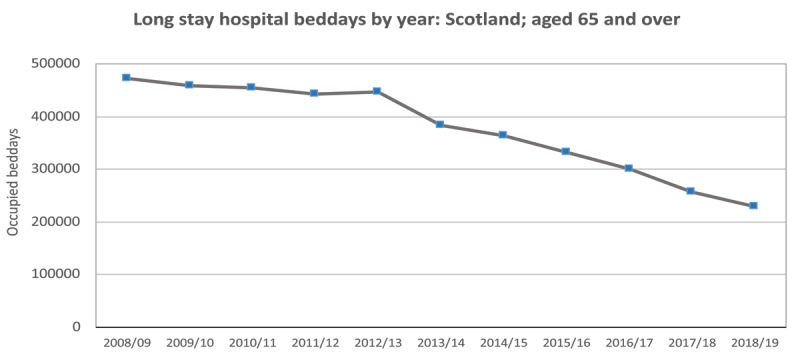
Long stay hospital beddays used by people aged 65+, 2008/09 to 2018/19. Source: SMR01E (PHS); based on discharges before Nov. 2020; people admitted originally as an emergency.

The number of people aged 65+ in long stay hospital care has markedly declined since 2008/09 and their beddays used reduced from 472,000 in 2008/09 to 229,000 in 2018/19 (***Figure 3***).

**Figure 4:**
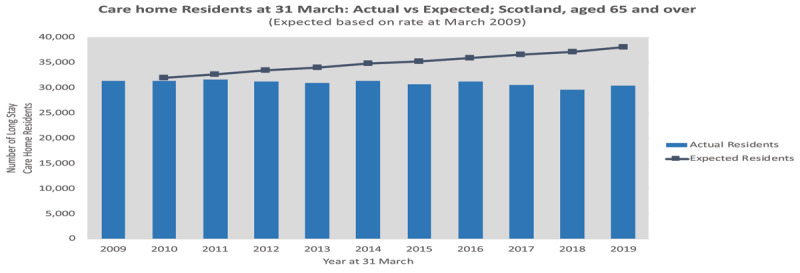
Long-Stay Care Home Residents aged 65+, 2009 to 2019: actual vs expected. Source: Scottish Care Home Censuses; NRS population estimates.

When days spent in long stay hospital care are combined with acute hospital stays, the actual bed days for people aged 65+ in 2018/19 are 27% lower than the population adjusted trend would have suggested. Notably, this gap between actual and expected has not been accompanied by an expansion in long term residential care [88]. The same comparative approach reveals the gap between actual and expected numbers of long-term care home residents has also grown – the number of age 65+ long-stay residents at March 2019 (30,418) is 20% lower than would have been expected based on 2009 rates (***Figure 4***).

**Figure 5:**
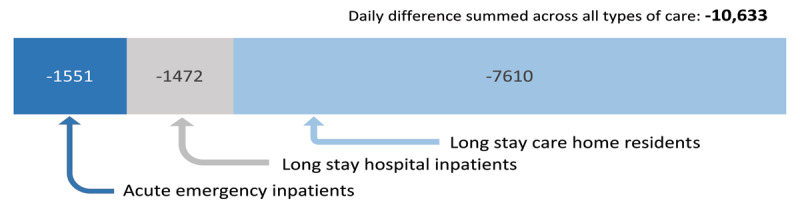
Institutional care avoided per day 2018/19: aged 65 and over. Data sources: SMR01, SMR01E, Care Home Census 2018/19 NRS population estimates.

The complexity and heterogeneity of integrated care make evaluation and attribution of economic impact difficult [89]. However, ***Figures 2***–***4*** demonstrate a significant shift to care at home, avoiding institutional care costs and releasing resource for investment in community health and care support and services. One way to appreciate the scale of the shift from the previous balance of care is to present the 2018/19 figures above as ‘daily averages’. This method suggests around 10,000 more people aged 65+ were living at home each day in 2018/19 than ‘expected’ (***Figure 5***).

Allied with a 6% reduction in emergency bed days for all adults (2014/15 – 2018/19), this shift has enabled a managed and proportionate disinvestment in hospital bed capacity while bed occupancy remains stable [87].

Multiple complex interventions have contributed to achieving this shift [31]. Evidencing the contribution of specific interventions is not an exact science. Routine national collection of data has been principally focused, to date, on acute healthcare and, more recently, has included linkable data from statutory social care services [90]. The impact of community interventions such as intermediate care, reablement and third sector support for wellbeing has been more difficult to assess at a national level. The full potential of linkable information from routinely collected general practice data has yet to be realised in Scotland.

One notable exception is availability of data on Anticipatory Care Planning (ACP), a person-centred approach that encourages practitioners to work with patients, carers and families to express their preferences and goals for future care. From 2008, national support for ACP built on innovation in a single General Practice [91] and has incrementally enabled spread to cover 5% of the population by February 2020. This focus on ACP has contributed to a modest increase in the time people spend at home or in a community setting in the last six months of life [92,93]. Following further rapid scale up during the Coronavirus pandemic, by October 2020 around 20% of primary care clinical records included an electronic summary of the patient’s anticipatory care plan that is routinely shared with out of hours and acute care providers.

**Figure 6:**
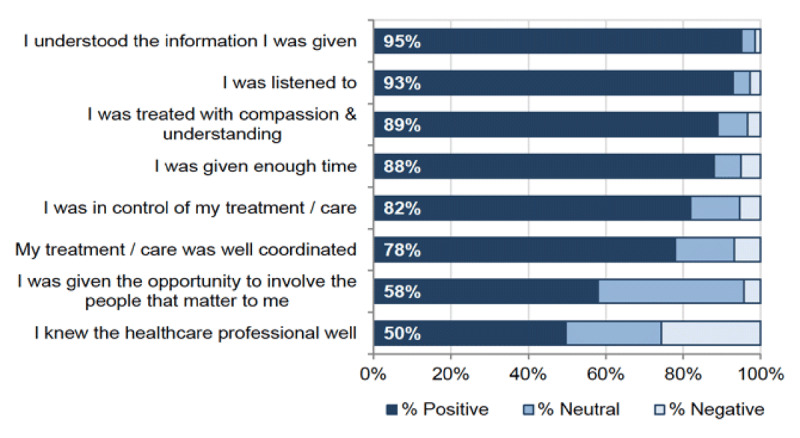
Scottish Government Health and Care Experience report 2017/18.

The value of qualitative information on outcomes for people, families and communities is widely recognised but is not easily tracked at a national level. Academic evaluations of specific initiatives, for example of the Links worker programme [66], the House of Care early adopters [67] and of a compassionate community in one IA [94], highlight many examples of improved personal, relational and community outcomes. A standardised national survey of Health and Care Experience is sent every two years to a random sample of citizens [95]. It seeks to capture their experiences of accessing and using local healthcare services and of receiving care, support and help with everyday living and caring responsibilities. Data from the 2017/2018 Primary Care Health and Care Experience survey suggests good levels of communication but a need for greater continuity and coordination of primary care (***Figure 6***).

In the most affluent areas of Scotland men experience 23.8 more years of good health and women and additional 22.6 years compared to the most deprived areas [96]. Integration of health and social care has had little impact on these systemic inequalities.

## Discussion

While few disagree with the vision for integration, national progress has often seemed slow and piecemeal [39,42,43,44,64]. As implementation requires careful alignment of many policies and support mechanisms with significant interdependencies, it is not surprising that there has been an ebb and flow of progress over time and across the country. One factor has been significant turnover in the first cohort of Chief Officers and IJB Chairs and by changes in key personnel in Ministerial, policy and senior NHS management posts [43,44].

Implementing major reform after a prolonged period of austerity has proven challenging. Now the economic context is even more volatile and uncertain as a result of Coronavirus. However the pandemic has undoubtedly demonstrated what can be achieved by working together across organisational and sectoral boundaries: better local collaboration, greater ability to pivot and enhanced capability to facilitate key infrastructure and practice changes at unprecedented speed. Facing stark health and economic challenges from the pandemic [97], IAs and NHS Boards must further strengthen their alliances with community partners and the third sector to improve lives and opportunities through a stronger focus on prevention, early intervention and targeted action on the wider determinants of health.

Progress on addressing inequalities in Scotland has been elusive, in common with many countries [81]. Relative inequalities have widened over the last 10 years and life expectancy at birth remains relatively poor and has not improved since 2012. Some argue this requires reimagining local governance beyond health and social care [98]. Although there seems little appetite for further structural change in Scotland’s public bodies, there is growing recognition of a need to strike the right balance between centrally directed organisations such as the NHS and flexible arrangements for local delivery through strong horizontal integration with community partners. A Blueprint document from local government [99] affirms the need to accelerate progress on integration and includes proposals for the next phase of reform. Several strategic actions are already underway, including the introduction of legislation to adopt the European Charter for Local Self Government [100] in Scots’ law. This aims to guarantee political, administrative and financial independence for Local Authorities along with new powers to raise and set taxes and make spending decisions based on local priorities and economic realities.

Linked with this is a commitment to reinvigorate the relationship between Public Health and Local Government to improve and protect community wellbeing, particularly for vulnerable populations who have experienced greater disadvantage from the health and economic impacts of Covid-19. Public Health Scotland’s Strategic Plan 2020–2023 sets out four priority areas of action where collaboration with local government on wider determinants of health will be critical [101]. These priorities are: Covid-19 response, recovery and renewal; understanding and influencing the economic, social and emotional factors that create good relationships and mental wellbeing, and eliminate discrimination and stigma; use of data and intelligence to understand the unique needs of Communities and Place to improve health and wellbeing in communities that experience the worst outcomes; and identifying and supporting evidence based actions to address poverty and improve child health.

## Lessons learned

Various published insights on Scotland’s integration story describe critical issues of leadership, culture, workforce, difficulties with demonstrating impact and managing a challenging financial context [22,31,39,44,64,65,102,103]. From analysis of these reports and reflecting on our accumulated wisdom as expert stakeholders collaborating in developing and implementing this policy reform for over a decade, we offer five key lessons for other systems considering similar reform:

1. **Engage and Involve:** Start with the ‘why’ and co-produce a compelling vision about improving lives and creating a better, more sustainable future. You are more likely to build a successful movement for change if you involve people who use and deliver services in co-designing the future. Bringing people together as equal partners is necessary to understand differing perspectives and challenges and creates an environment where all concerned may be more open to being influenced. Realistic conversations on the values and outcomes that matter to individuals and communities are seen as increasingly important. Touch hearts and minds with judicious use of information and stories about the quality and experience of care.
2. **Empower and Enable:** Every community is different and, perhaps curiously, many are more likely to be open to new ways of working from another country than their neighbouring district. Transferring solutions, no matter how well tested, will fail if implemented without due regard for local culture, history and buy in. Understanding the local context and readiness for change is important so invest time in building trusting relationships. Strive to understand what underpins different behaviours and levels of co-operation. Be prepared to cede power and control to partners, individuals and communities to empower and enable them to design local and sustainable solutions.
3. **Collaborate and Coordinate:** Successful transformation requires coordinated efforts across the whole of government, the whole of the health and care system at every level, and with citizens. Even with political will, new legislation is not enough to deliver radical reform. The reasons are complex and relate to issues of power and vested interests, and the inherent management and leadership capability for managing complex change. Multiple policy, financial and contractual levers need to be aligned, mutually reinforcing and their deployment well timed and coordinated to create the right conditions for integration to flourish.
4. **Innovate and Improve:** Adopting technology and new ways of working is complex. In ordinary circumstances, changes will take longer than one might reasonably expect but in crisis situations the pace and scale of change may be transformational. Be ready when opportunities present themselves. Embrace disruption and external challenges as opportunities for innovation to overcome barriers and change pace. Invest in improvement capacity and build local capability to test, spread and scale up new ways. Whenever possible look to co-produce solutions with citizens and with the workforce. People, not policies, change lives. Culture change can take a generation so invest from the outset in developing the habits of people centred integrated care within undergraduate training curricula and postgraduate practice.
5. **Reflect and Learn:** New ways will only make sense if anticipated improvements can be evidenced and unintended consequences minimised. Some things may appear to get worse before they get better. Do not stint on analytical support and invest in formative evaluation from the outset. Crucially, be humble and curious and learn from your mistakes and from other systems on a similar journey. It may take some time to fully understand the impact of integration but stick with it – this is a marathon not a sprint.

## Conclusions

Published reports note positive progress in collaborative working and high level national data demonstrate encouraging evidence of impacts from this policy reform. Only time will tell if the potential can be fully realised in order to scale up and sustain these early gains. Notably, even ‘standing still’ in performance over the last five years has been a significant achievement given the economic challenges, increasing system demands and complexity of care needs. However, important questions remain unanswered. Could the early progress achieved through the RCOP programme [30] have been sustained and spread to other care groups without introducing new legislation? Is there any difference between the outcomes realised through the two models of integration (Lead Agency vs Body Corporate)? While there has been national scrutiny of progress [28,43,44], there has been little in-depth academic research on observed changes in local processes, relationships and cultures over time.

Experience from Covid-19 to date has demonstrated what can be achieved by working together across organisational and sectoral boundaries: better local collaboration, greater ability to pivot and enhanced capability to facilitate key infrastructure and practice changes at unprecedented speed. Careful reflection and analysis of this experience will be required to understand why this was the case and what were the respective contributions of people, communities, processes, structures and technologies in creating the conditions to enable rapid change. The Researcher in Residence model [104] could be a very useful vehicle for rapid, real-time and action-orientated research to understand the important opportunities for transformation in this turbulent period [105]. This insight will be critical to strengthen alliances with community partners for population health to improve lives and opportunities for all.

In unprecedented times and in an uncertain and rapidly evolving political, economic and health and social care landscape, there is one certainty: Scotland’s integration story will continue to unfold.
